# Temporal dynamics and species-level complexity of *Prevotella* spp. in the human gut microbiota: implications for enterotypes and health

**DOI:** 10.3389/fmicb.2024.1414000

**Published:** 2024-07-09

**Authors:** Na Han, Xianhui Peng, Tingting Zhang, Yujun Qiang, Xiuwen Li, Wen Zhang

**Affiliations:** National Key Laboratory of Intelligent Tracking and Forecasting for Infectious Diseases, National Institute for Communicable Disease Control and Prevention, Chinese Center for Disease Control and Prevention, Beijing, China

**Keywords:** microbiome, *Prevotella* spp., human gut, enterotypes, genome

## Abstract

The concept of “enterotypes” in microbiome research has attracted substantial interest, particularly focusing on the abundance of *Prevotella* spp. in the human gut. In this study, the intricate dynamics of *Prevotella* spp. in the human gut microbiota was investigated, based on the metagenomic method. First, 239 fecal samples from individuals across four regions of China revealed a bimodal distribution, highlighting the abundance and variability in *Prevotella* spp. within the Chinese population. Second, the longitudinal cohort study included 184 fecal samples from 52 time points collected from seven individuals who demonstrated either the outbreaks or disappearances of *Prevotella* spp., emphasizing the transient nature of *Prevotella* abundance levels and suggesting shifts in *Prevotella* “enterotypes.” Furthermore, a turnover of the dominant *Prevotella* spp. was observed, indicating the potential presence of diverse subtypes of *Prevotella* enterotype. Notably, the genomic analysis demonstrated the persistence of specific *Prevotella* strains within individuals over extended periods, highlighting the enduring presence of *Prevotella* in the human gut. In conclusion, by integrating the temporal and geographical scales in our research, we gained deeper insights into the dynamics of *Prevotella*, emphasizing the importance of considering the dynamics at the time and species level in gut microbiota studies and their implications on human health.

## Introduction

The characterization of the gut microbiota has sparked debate, particularly regarding the concept of “enterotypes” (Arumugam et al., 2011), akin to blood types, suggesting distinct gut microbiota types that could be correlated with overall health (Siezen and Kleerebezem, 2011; Wu et al., 2011; Costea et al., 2018). *Prevotella*, one of the most prevalent genera in the gut, is a critical type in these distinct enterotypes (Arumugam et al., 2011; Siezen and Kleerebezem, 2011; Wu et al., 2011; Costea et al., 2018). *Prevotella* has been the subject of extensive research because of its complex relationship with human health (De Filippis et al., 2019; Metwaly and Haller, 2019; Abdelsalam et al., 2023). *Prevotella* has been associated with positive health outcomes, promoting the fermentation of dietary fiber and the production of short-chain fatty acids (Bedarf et al., 2017). These fatty acids have been linked to improved metabolic health and reduced risk of certain diseases, such as inflammatory bowel disease (Bajer et al., 2017). *Prevotella* has also been linked to a balanced immune response and maintenance of gut barrier function, both of which are essential for overall health. On the other hand, an overabundance of *Prevotella* spp. in the gut has been associated with negative health outcomes, including Parkinson’s disease (Stefanis, 2012), and certain types of cancer (Gunathilake et al., 2019). Some studies have also suggested that *Prevotella* spp. may contribute to the development of obesity (Moran-Ramos et al., 2023) and metabolic disorders by promoting carbohydrate fermentation and the production of potentially harmful metabolites (Metwaly and Haller, 2019).

The conflicting research findings on the impacts of *Prevotella* spp. on human health underscore the critical need for further investigation to enhance our comprehension of its role within the gut microbiota and its implications on human health. The latest taxonomic findings offer a potential explanation for the intricacies of *Prevotella*. These recent taxonomic studies from the International Journal of Systematic and Evolutionary Microbiology (Oren and Göker, 2023) have reclassified the genus *Prevotella* into seven distinct genera, introducing four novel genera: Segatella, Hoylesella, Leyella, and Palleniella (Hitch et al., 2022). This new classification hints at the diverse relationships that different microbes under the original *Prevotella* classification may have with human health, necessitating a more nuanced approach to research.

However, previous analyses of gut microbiota structures have predominantly focused on the level of the original *Prevotella* genus. There has been a lack of resolution at the species level and neglect of research on the distribution patterns of the various taxa within the updated *Prevotella*. Consequently, there has been a dearth of in-depth investigations linking the latest taxonomic insights with outcomes in gut microbiota. Numerous inquiries persist regarding the existence of *Prevotella* enterotypes, the potential for additional subtypes, whether they consist of multiple species or a select few key species, the interrelationships among species across the seven *Prevotella* genera, their stability within the human body, and the underlying reasons for any fluctuations.

Drawing on this taxonomic framework, we have the opportunity to reassess the concept of *Prevotella* enterotypes through the use of temporal and geographical scales, thereby corroborating its presence and impact on human health in the Chinese population.

## Materials and methods

### Sample collection and cohort description

As a part of the Chinese Microbiome Project (CMP), 239 fecal samples were collected from individuals residing in four provinces across China: Beijing, Jiangsu, Henan, and Sichuan, representing diverse geographical regions within the country. Prior to the sample collection, the participants underwent a pretest and completed a comprehensive pre-questionnaire, which included demographic details, personal and familial medical histories, and lifestyle practices such as smoking, physical activity, and dietary patterns (Supplementary Table S1). These data were used to evaluate the general health status of the participants. Detailed information on the sampling procedure in the CMP can be found in our previous publication (Zhang et al., 2022).

In conjunction with the CMP initiative, fecal samples were procured monthly from seven healthy individuals residing in Beijing, China, spanning the timeline from March 2017 to October 2022. To ensure a longitudinal study, samples from individuals with fewer than 10 data points were excluded. During each sampling session, the participants completed a detailed questionnaire regarding their medical history over the preceding month, and physical metrics, including height, weight, blood pressure, and blood glucose levels, were documented on-site. Ultimately, 184 fecal samples were obtained from these 7 individuals, resulting in a dataset comprising 52 data points. The study was approved by the bioethical committee (ICDC-2022-001) of the Chinese Centre for Disease Control and Prevention (CDC), and all procedures were conducted per the relevant guidelines and regulations, with written informed consent obtained from all participants.

### DNA extraction, sequencing, and quality control

The fecal samples were collected within 24 h of completion of the pretest and pre-questionnaire using disposable bedpans, to minimize the risk of contamination from toilet water.

The DNA was extracted within 24 h of sample collection using the QIAamp Fast DNA Stool Mini Kit (Qiagen, Hilden, Germany) following the manufacturer’s instructions. Subsequently, shotgun metagenomic libraries were prepared using the TruePrep DNA Library Prep Kit from Illumina, and sequencing was performed on an Illumina HiSeq platform to generate paired-end reads of 150 bp. Quality control analysis involved the use of FastQC^1^ and Fastp (Chen et al., 2018) to filter out low-quality reads. This included trimming of low-quality bases (< Q20) and retention of reads with a length ≥ 100 bp. After the quality control of raw reads, the Bowtie2 tool was used to remove the human sequences (Langmead and Salzberg, 2012).

The public fecal microbiome data was downloaded from the Human Microbiome Project (HMP) database (Human Microbiome Project, 2012; Lloyd-Price et al., 2017) and it included shotgun metagenomics data from 153 healthy individuals.

### Metagenomic data analysis pipeline

The analysis pipeline utilized in this study is available on GitHub.^2^

The MetaPhIAn2 tool was used to calculate the relative abundance of *Prevotella* spp. for the qualified samples (Truong et al., 2015, 2017), and an evolutionary tree of *Prevotella* strains was constructed using StrainPhiAn tool (Truong et al., 2017). Another strain-level analysis software, SameStr (Podlesny et al., 2022), was used to determine whether any two positive samples contained the same strain. The metagenome bins were assembled using SPAdes v3.13.0 (Bankevich et al., 2012) and MetaBAT v2.12.1 (Kang et al., 2019), followed by taxonomic classification using the GTDB-Tk toolkit (Chaumeil et al., 2019, 2022). A phylogenetic tree was constructed using the ANIclustermap^3^ for the metagenome bins identified as *Prevotella,* based on the average nucleotide value, with the additional inclusion of 88 strain genomes from National Center for Biotechnology Information (NCBI). The final phylogenetic tree was annotated using the iTOL tool (Letunic and Bork, 2021). The identification of single nucleotide polymorphism (SNP) between the genomes was achieved using the MUMmer software (Marçais et al., 2018). The line, bar, and circular plots in this study were generated by R scripts using the ggplot2 package (Wickham, 2009). The co-occurrence network of the gut microbiota was constructed using the R package igraph (Csardi and Nepusz, 2006). The correlation between bacterial species was calculated using the Spearman algorithm. The correlations with a coefficient greater than 0.6 or less than −0.6, as well as a *p*-value of <0.001, were retained in the analysis. The enterotype of each sample was assigned using an online tool^4^ and R packages biotypes.^5^

## Results

### Abundance of *Prevotella* on genus and species level

**Figure 1:**
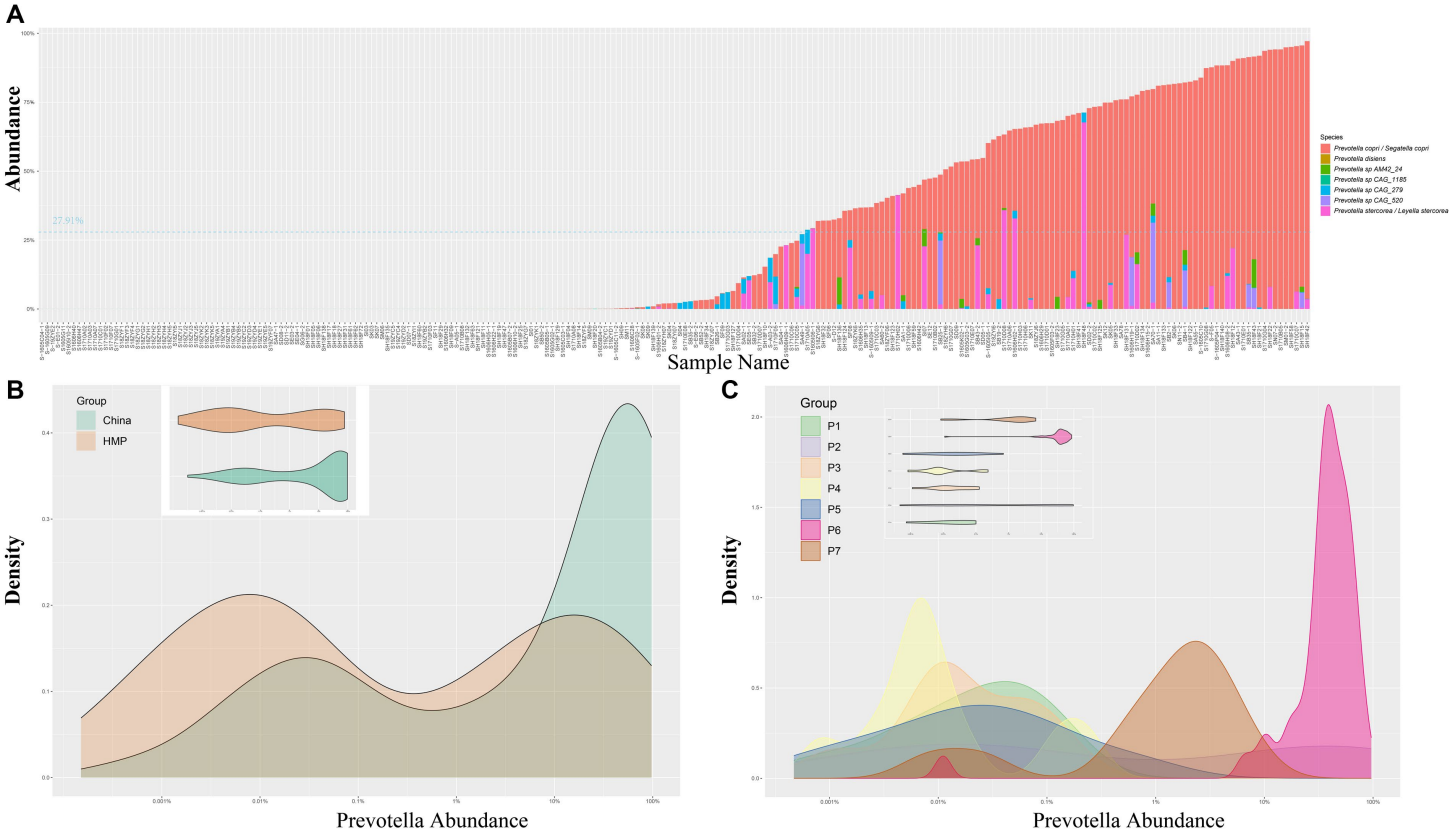
**(A)** Bar chart representing the abundance of *Prevotella* in 239 samples from the CMP dataset. **(B)** Density plot and violin plot for the abundance of *Prevotella* in the CMP and HMP datasets. **(C)** Density plot and violin plot displaying the distribution of *Prevotella* abundance in 7 individuals from the time cohort in this study. CMP, Chinese Microbiome Project; HMP, Human Microbiome Project.

To elucidate the structure of the gut microbiota and correlate the results with enterotypes, we conducted analyses using the original and the latest taxonomic information. Under the original classification framework, *Prevotella* was considered as a genus. Our findings revealed a wide variation in the abundance of *Prevotella* among individuals across China. While the average abundance of *Prevotella* was 27.91% according to the MetaPhIAn method, certain samples exhibited unexpectedly high levels of approximately 97.31% (Figure 1A). This distribution indicates a bimodal pattern of the abundance of *Prevotella* within the Chinese population, with some individuals showing high abundance while others showed low levels (Figure 1B). We utilized the same methodology to analyze the publicly available HMP database (Human Microbiome Project, 2012; Lloyd-Price et al., 2017). Similar patterns were observed in the analysis of the HMP public dataset, providing further evidence for the existence of a subset of individuals with elevated *Prevotella* levels in the gut microbiota.

Furthermore, we utilized the latest taxonomic classification to analyze the gut microbiota. Recent taxonomic research (2023) has subdivided the genus *Prevotella* into seven genera, introducing four new genera named Segatella, Hoylesella, Leyella, and Palleniella (Hitch et al., 2022). *Prevotella copri* was reclassified as *Segatella copri*, detected in 66.94% of the 239 samples, showing the highest detection rate (Figure 1A). *Prevotella stercorea* was reclassified as *Leyella stercorea*, detected in 25.10% of the 239 samples, ranking second. While other species were also detected (Figure 1A), their abundance and positivity rates were comparatively lower.

### Short-term outbreaks or disappearances of *Prevotella* over time

**Figure 2:**
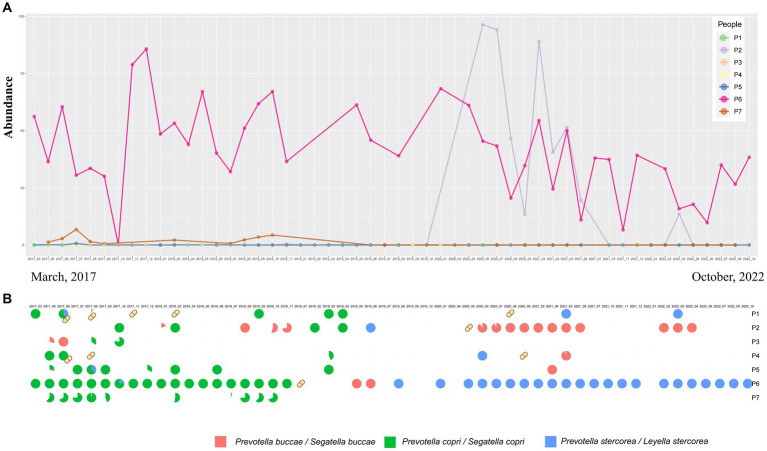
**(A)** Genus-level abundance of *Prevotella* from samples at different time points sourced from seven different participants. **(B)** Temporal changes in the proportion of abundance of *Prevotella* at the species level over time. Yellow capsule icons represent participants who were on antibiotics during that month.

In our study, we further investigated the temporal dynamics of *Prevotella* species under both the original and the latest taxonomic frameworks. In the original classification framework, the longitudinal study of up to 5 years demonstrated short-term blooms or decreases in the abundance of *Prevotella* over time (Figures 1C, 2A). Short-term blooms, defined as instances where the abundance of *Prevotella* exceeded 5 times the individual’s average abundance, and short-term decreases, defined as abundance dropping to more than 5 times lower than the previous time point (Zhang et al., 2022; Han et al., 2024), were observed. These fluctuations were recorded at 17 time points over the 5 years and were distributed among 7 individuals (Supplementary Figure S1). In the two instances of short-term blooms, the participants reported antibiotic drug usage, while there were no reports of antibiotic use related to the other fluctuations or time points. This suggests that high or low *Prevotella* abundance may only be temporary, indicating potential variability in *Prevotella* “enterotypes” based on single time point samples. Under the latest taxonomic framework, our findings similarly support the occurrence of short-term blooms or decreases in abundance for various *Prevotella*/Segatella/Leyella species (Supplementary Figure S1).

### Dominant species turnover within the *Prevotella* genus

The definition of “dominant species” in this study is as follows: within the samples if only one species of *Prevotella* is detected, or if the abundance of this species exceeds 80% of the total abundance of all species within the same genus, then this is referred to as the dominant species.

*Prevotella copri* (*Segatella copri*) was the most abundant species in the CMP cohort study and was identified as the dominant species in 51.46% of samples (Figure 1A). Other species such as *Prevotella stercorea* (*Leyella stercorea*) were also identified as dominant species in 5 samples (2.09%). The results obtained from the time dataset also show that *Prevotella copri* (*Segatella copri*) is the most prevalent among all *Prevotella* species. *Prevotella copri* (*Segatella copri*) was detected in 53 of 240 samples, and it was the dominant species in 34 samples. However, changes in prevalence were observed over time (Supplementary Figures S1A–F). Furthermore, there was a turnover of the dominant *Prevotella* species over time, indicating that *Prevotella* not only changes in abundance but also exhibits turnover at the species level (Figure 2B). Upon reviewing the responses to the questionnaire regarding antibiotic use during each sampling, we found a correlation between antibiotic usage and the turnover of dominant species (Figure 2B). For instance, in the case of individual P6, who had a record of antibiotic use in January 2019, the dominant *Prevotella* species shifted from *Prevotella copri (Segatella copri)* before antibiotic treatment to *Prevotella buccae (Segatella buccae)* after treatment. However, antibiotic use alone could not explain the turnover of all the dominant species. For the same individual, after July 2019, the dominant species reverted to *Prevotella stercorea* (*Leyella stercorea*) without any antibiotic usage. These results suggest that besides antibiotic use, other unknown factors also play a role in driving changes in *Prevotella* within the human gut.

### Persistence of *Prevotella* at the strain level in the human gut

**Figure 3:**
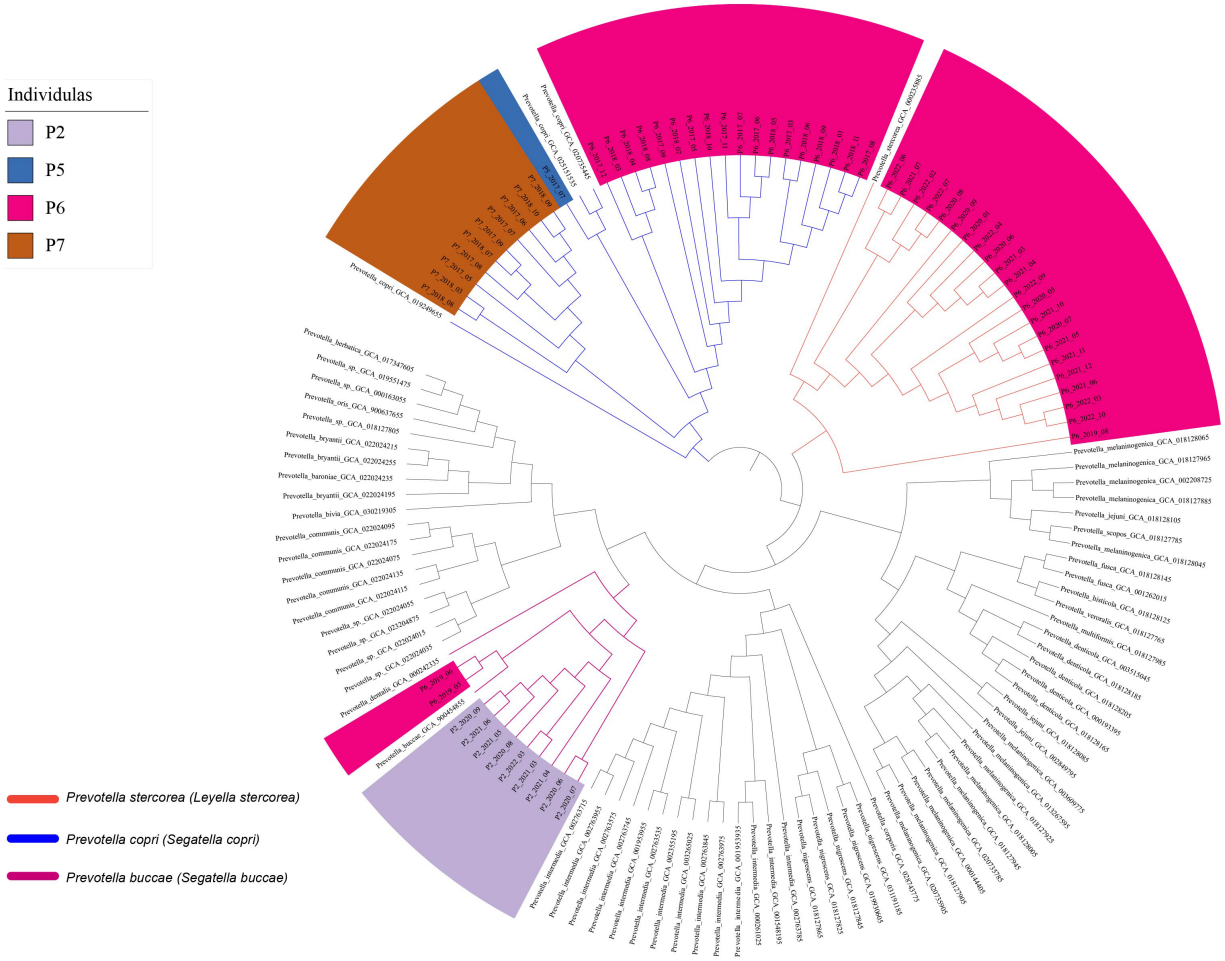
The phylogenetic tree based on genomes.

At the strain level, genome analysis revealed relatively stable *Prevotella* strains within samples from the same individual over time. This was corroborated by the consistent clustering of *Prevotella copri* (*Segatella copri*) strains from individual P6 across multiple time points (March 2017 to November 2018), and the genomic similarity (average nucleotide identity value) between these strains was higher than 99.95%, implying a degree of strain persistence within the gut microbiota (Figure 3). *Prevotella stercorea* (*Leyella stercorea*) was also identified in the samples collected at multiple time points from August 2019 to October 2022 from individual P6; it exhibited high similarity at the genome level, indicating its persistence within the individual at the annual level. Similar characteristics were observed for *Prevotella buccae* (*Segatella buccae*) from individual P2 and *Prevotella copri* (*Segatella copri*) from individual P7, confirming the persistence of specific *Prevotella* strains within individuals over time. These findings highlight the enduring presence of *Prevotella* strains in the human body over an extended period.

We further evaluated the single nucleotide polymorphisms (SNPs) between strains at the genome level at different time points. Despite a high degree of similarity among the strains (ANI > 99.95%, Figure 3), we observed tens to hundreds of single nucleotide polymorphisms (SNPs) at the genome level between pairs of the genome (Avg: 0.1 SNP/kb). By examining the temporal distance between the sampling points of the samples and the distribution of SNPs (Supplementary Figure S3), we did not find a significant correlation between the number of SNPs and time and distance. These results indicate that the majority of SNPs among *Prevotella* strains in the human gut are neutral mutations, rather than being fixed over time within the human gut *Prevotella* population.

In addition to genomic analysis, marker genes from each species were utilized to construct a phylogenetic tree (Supplementary Figure S2) and SameStr software was used to analyze whether they originated from the same strain. It is worth noting that the marker genes of these species were sourced from the Metaphlan2 database (Truong et al., 2015, 2017). The results, as shown in Supplementary Figure S2, are consistent with the genomic analysis (Figure 3), supporting the notion that the same strain can exist within an individual for a certain period, up to years. Replacement of *Prevotella* strains within individuals in adjacent months was not observed in this study.

### Relationship between *Prevotella* and other microbes

**Figure 4:**
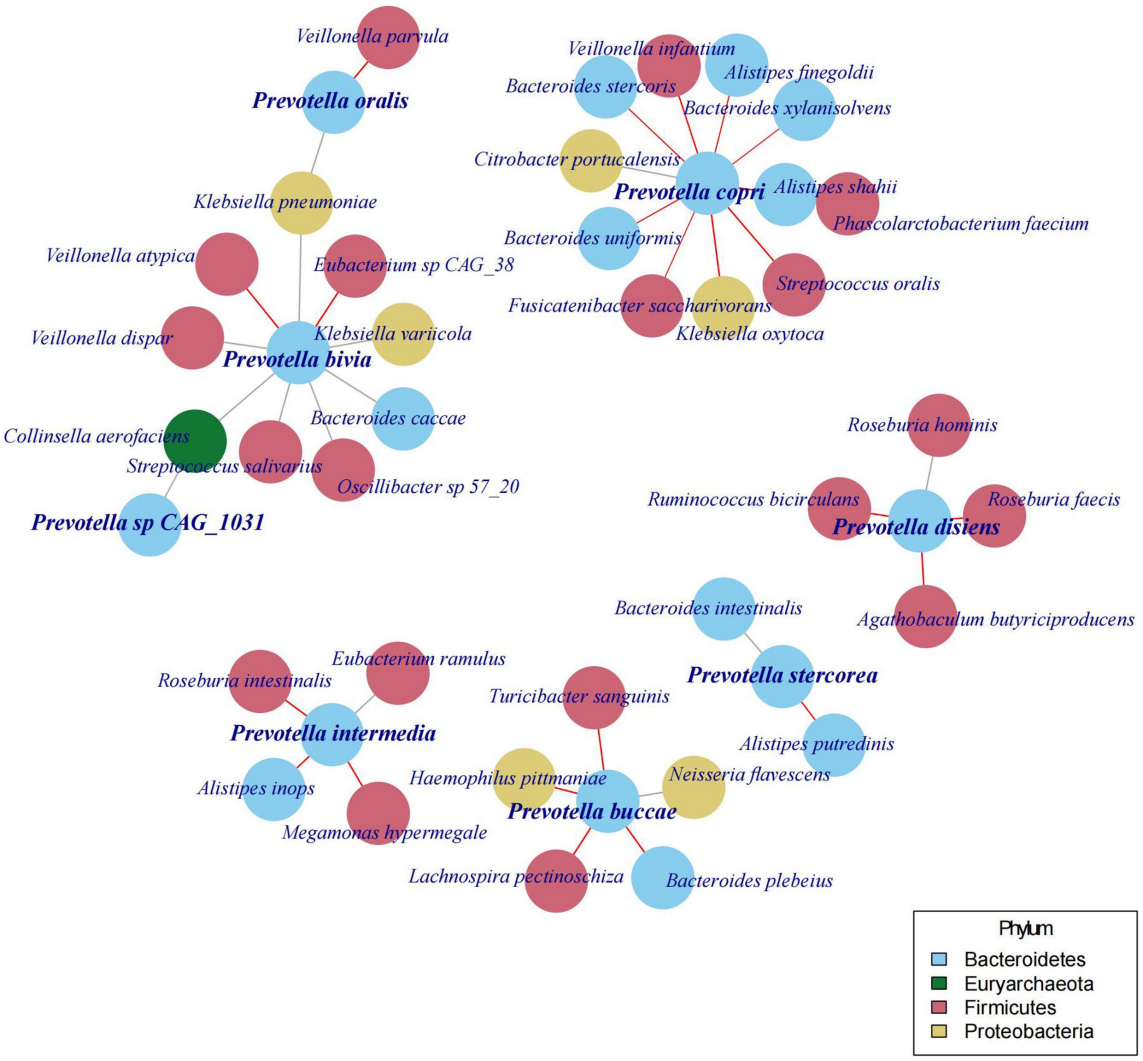
The co-occurrence network of *Prevotella* species with 37 other bacterial species.

In the preceding sections of this study, we discovered various species of *Prevotella* existing in the human gut, and their abundance is not stable over time and may even show a turnover of the dominant species. In the following analysis, we aimed to determine whether changes in the abundance and the species of *Prevotella* would also lead to variations in other gut microbial species. Therefore, we constructed a co-occurrence network between *Prevotella* and other bacteria based on the community structure of gut microbiota and found that the abundance of *Prevotella* species is correlated with 37 other bacterial species (Figure 4).

Different *Prevotella* species are associated with distinct sets of other bacterial species. For instance, *Prevotella copri (Segatella copri)* is linked to 11 other species, with a positive correlation with one species (*Citrobacter portucalensis*) and a negative correlation with 10 species. Conversely, *Prevotella stercorea (Leyella stercorea)* is only associated with 2 other species, and these associated species are different from those linked to other *Prevotella* species. Moreover, the bacterial species associated with *Prevotella copri (Segatella copri)* are distinct from those associated with *Prevotella bivia, Prevotella buccae, Prevotella disiens, Prevotella intermedia,* and *Prevotella oralis*. Although further investigations are required to confirm the causal relationships between these bacterial species, the above findings underscore the diversity among the various species of *Prevotella*.

## Discussion

In recent years, the concept of “enterotypes” has garnered attention in microbiome research, proposing distinct gut microbiota compositions based on the abundance of certain bacterial genera, including *Prevotella* (Arumugam et al., 2011; Siezen and Kleerebezem, 2011; Wu et al., 2011; Costea et al., 2018; Metwaly and Haller, 2019; Couch et al., 2021; Alili et al., 2022; Fu et al., 2022; Liang et al., 2022; Moran-Ramos et al., 2023). However, “enterotypes” have faced challenges and criticisms. This study, based on a comprehensive survey, confirmed that *Prevotella* abundance in the human gut occurs in two distinct groups, with some individuals in the population experiencing a high abundance while others experience a low abundance. Furthermore, the longitudinal analysis revealed that the abundance of *Prevotella* is not constant, with sudden increases or decreases observed in samples from the same individual at specific time points, which suggests that single time point assessments may capture transient fluctuations in the abundance of *Prevotella*, potentially leading to false classifications for its “enterotype.” This variability underscores the complexity of microbiome studies. Although the results of this study do not definitively confirm or refute the existence of a *Prevotella* enterotype, they emphasize the importance of considering temporal dynamics when evaluating the characteristics of gut microbiota.

Moreover, the relationship between *Prevotella* spp. and health needs to be evaluated at a finer level as previous studies (Zhang et al., 2019; Fu et al., 2022; Liang et al., 2022) are often based on genus-level surveys, particularly through 16S sequencing analysis, and have not fully indicated the presence of multiple *Prevotella* species in the gut microbiota, each potentially serving as a dominant species. This study revealed that even samples with similar abundances of *Prevotella* could harbor different species, with the turnover and replacement of dominant species observed in samples from the same individual at different time points (Figures 2A,B). In this research, we also confirmed that with the variation in different *Prevotella* species, the associated gut microbiota differed, highlighting the diversity among *Prevotella* species. These findings imply that the samples with a similar abundance of *Prevotella* may elicit different interactions with the host owing to the presence of distinct species. Therefore, if an enterotype does exist, it likely encompasses multiple subtypes at the species level, necessitating a more nuanced approach when studying the association between gut microbiota and human health, focusing on the species level. The latest taxonomic research (2023) further subdivided the genus *Prevotella* into seven genera, including four novel genera for which the names *Segatella, Hoylesella, Leyella*, and *Palleniella* are proposed (Hitch et al., 2022). According to this updated classification framework, our study confirms that *Prevotella copri (Segatella copri)* is the most abundant and widely distributed species among the mentioned taxa, serving as the dominant species in 51.46% of samples in the CMP dataset. Furthermore, other *Prevotella* species coexist in fecal samples, with some of these acting as dominant species in certain samples, such as *Prevotella stercorea (Leyella stercorea)* and *Prevotella buccae (Segatella buccae)*. Furthermore, our study validates the temporal turnover among these species at the time scale (Figure 2).

Although a turnover was observed in the dominant *Prevotella* species and sudden fluctuations in their abundance, our investigation demonstrated that *Prevotella* strains can persist in the human body for an extended period. The 5-year longitudinal study revealed that *Prevotella copri (Segatella copri)* was consistently present in samples from P6 (March 2017 to November 2018), with genomic analysis indicating the same strain, without a turnover at the strain level. This finding is in contradiction with our previous finding based on *Escherichia coli* (Han et al., 2024), as this species experiences a rapid monthly turnover rate (87.5% within a month), whereas *Prevotella* persists in the gut for a longer duration. We refer to *Escherichia coli* and other rapidly turning over bacteria, similar to it, as transient gut microbes. In contrast, for *Prevotella*, which can persist in the gut for a certain period, we are inclined to believe they can establish colonization within the gut. In this study, we also observed the evolutionary trajectory of *Prevotella* colonization in the gut. Over the years of persistence of *Prevotella* in the human gut, we did not detect a rise in the number of SNPs with prolonged colonization. This observation implies the potential predominance of neutral evolution within *Prevotella*’s evolutionary trajectory in the human gut.

In summary, our investigation of *Prevotella* spp., an important component of the gut microbiota, confirmed sudden fluctuations in abundance at the genus level, turnover of dominant species at the species level, and the persistence of the same strain for years. We gained deeper insights into *Prevotella* by combining the temporal and geographical scales. This underscores the need to consider the dynamics at the time and species level when studying the gut microbiota and its implications on human health. Further research is warranted to elucidate the complex interactions of *Prevotella* with the host, as well as the factors (lifestyles, age, and drug usage) influencing its abundance and activity in the gut, ultimately advancing our understanding of the role of *Prevotella* on human health.

## Data availability statement

The datasets presented in this study can be found in online repositories. The names of the repository/repositories and accession number(s) can be found at: https://www.ncbi.nlm.nih.gov/sra/PRJNA1116923, PRJNA1116923.

## Ethics statement

The studies involving humans were approved by our analysis involved fecal samples obtained from healthy Chinese individuals who provided written informed consent prior to enrolment in this study. Furthermore, the study was approved by the China CDC, and all experiments were performed in accordance with relevant guidelines and regulations. The studies were conducted in accordance with the local legislation and institutional requirements. The participants provided their written informed consent to participate in this study.

## Author contributions

NH: Data curation, Investigation, Methodology, Software, Writing – original draft. XP: Formal analysis, Software, Writing – review & editing. TZ: Data curation, Software, Visualization, Writing – review & editing. YQ: Investigation, Validation, Writing – review & editing. XL: Investigation, Validation, Writing – review & editing. WZ: Methodology, Writing – original draft, Writing – review & editing.

## Footnotes

^1^ https://www.bioinformatics.babraham.ac.uk/projects/fastqc/

^2^ https://github.com/zhangwencdc/*Prevotella*

^3^ https://github.com/moshi4/ANIclustermap

^4^ https://enterotype.embl.de/

^5^ https://github.com/tapj/biotyper
