# Supplementary figures and images for: Temporal dynamics and species-level complexity of Prevotella spp. in the human gut microbiota: implications for enterotypes and health
Source: Front Microbiol. 2024 Jul 9;15:1414000. doi: 10.3389/fmicb.2024.1414000 (PMC11265296; doi:10.3389/fmicb.2024.1414000)

A

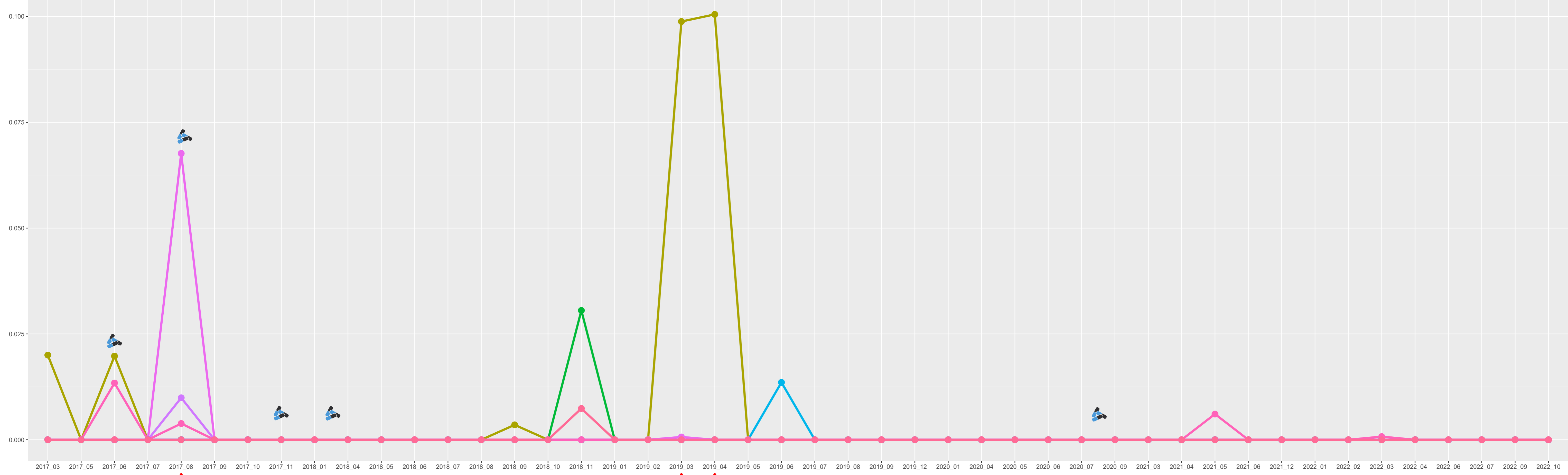

P1

B

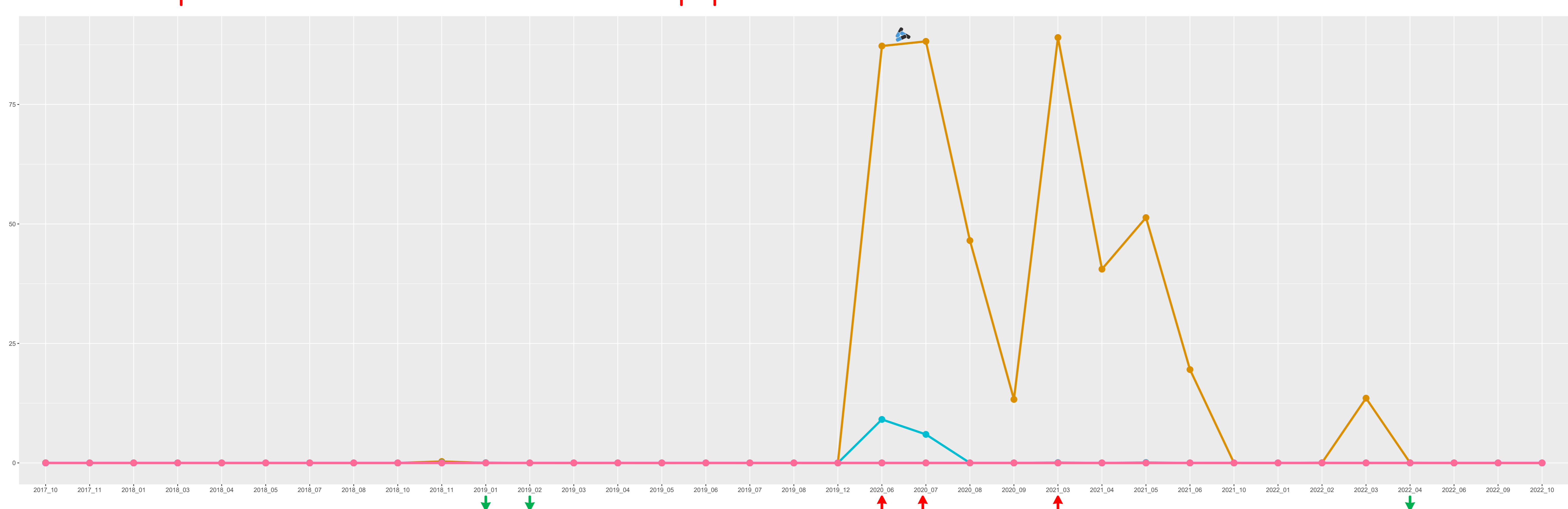

P2

C

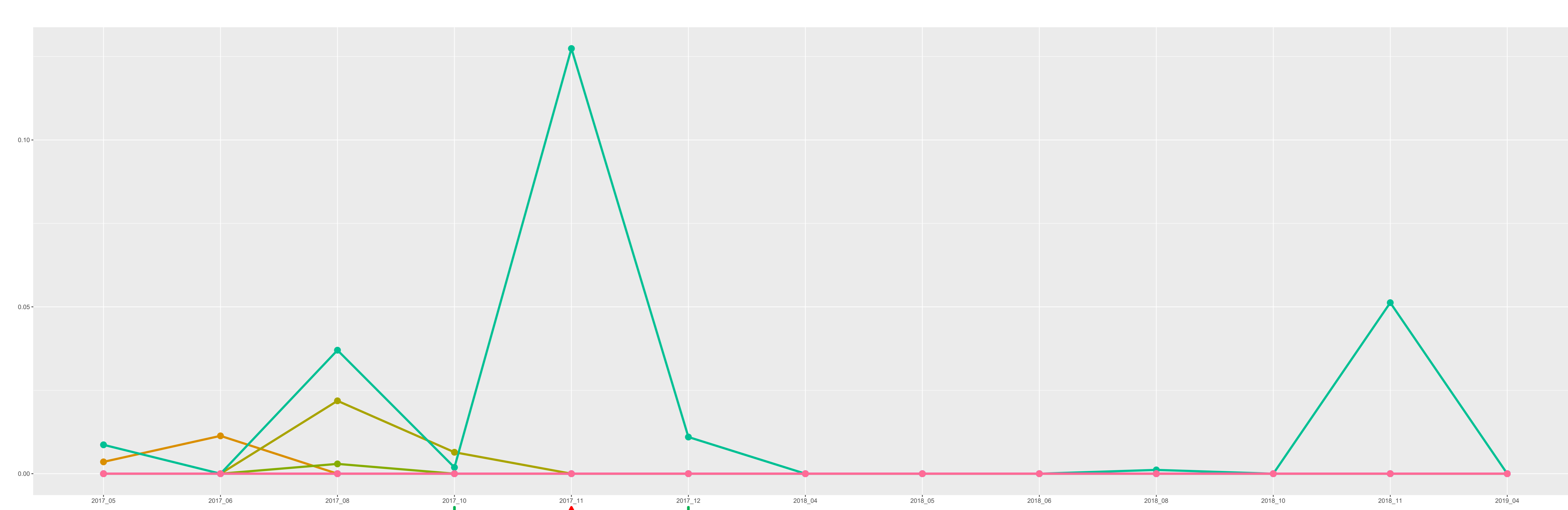

P3

D

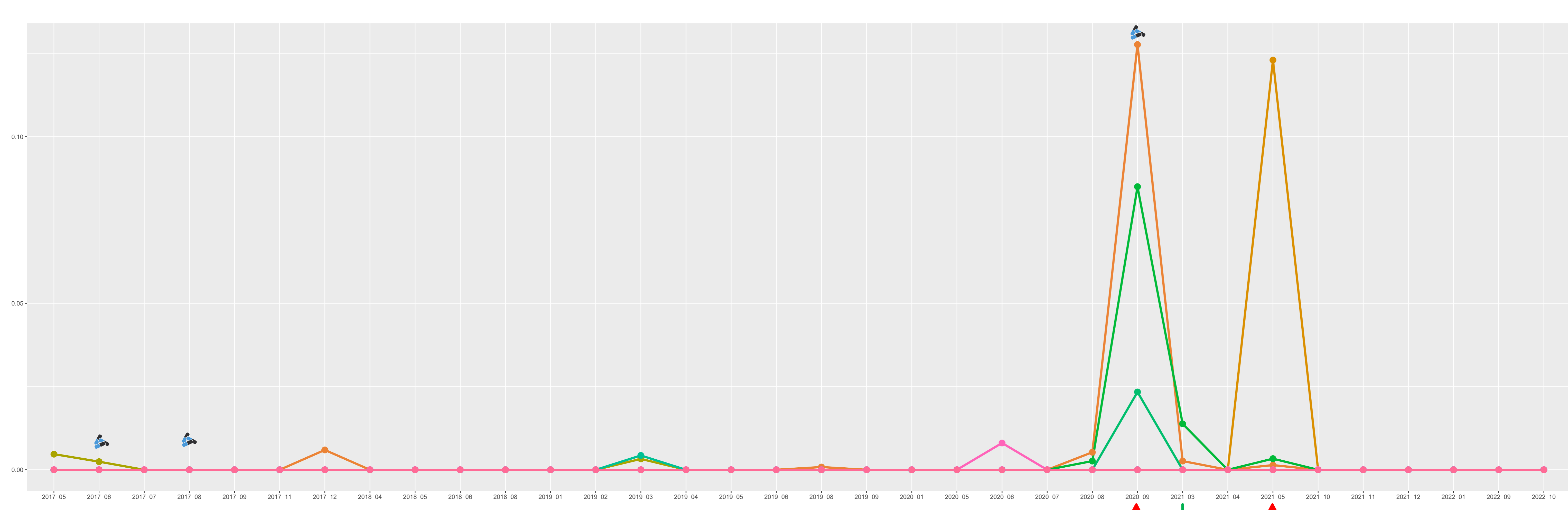

P4

E

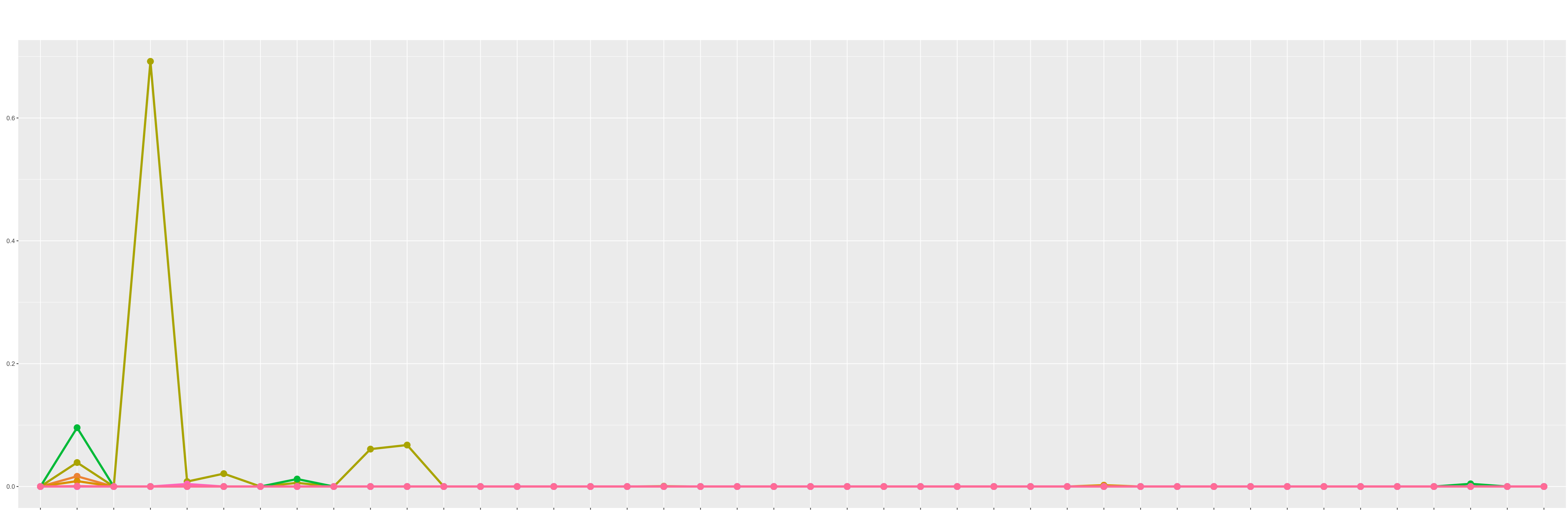

P5

F

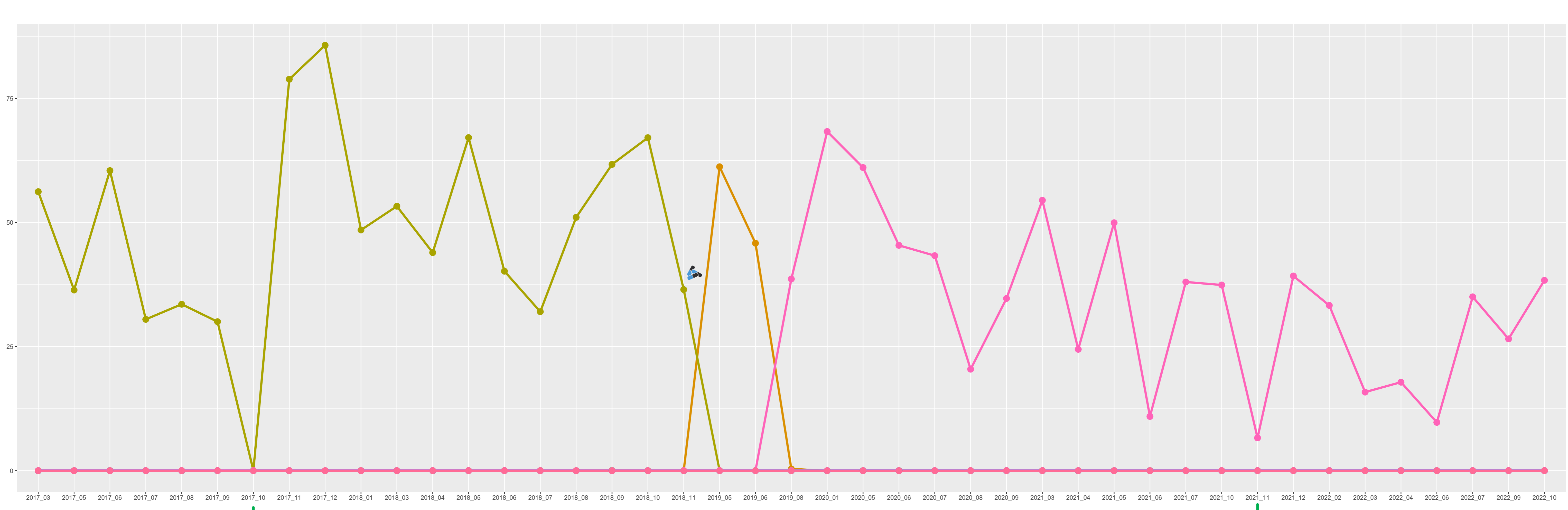

P6

G

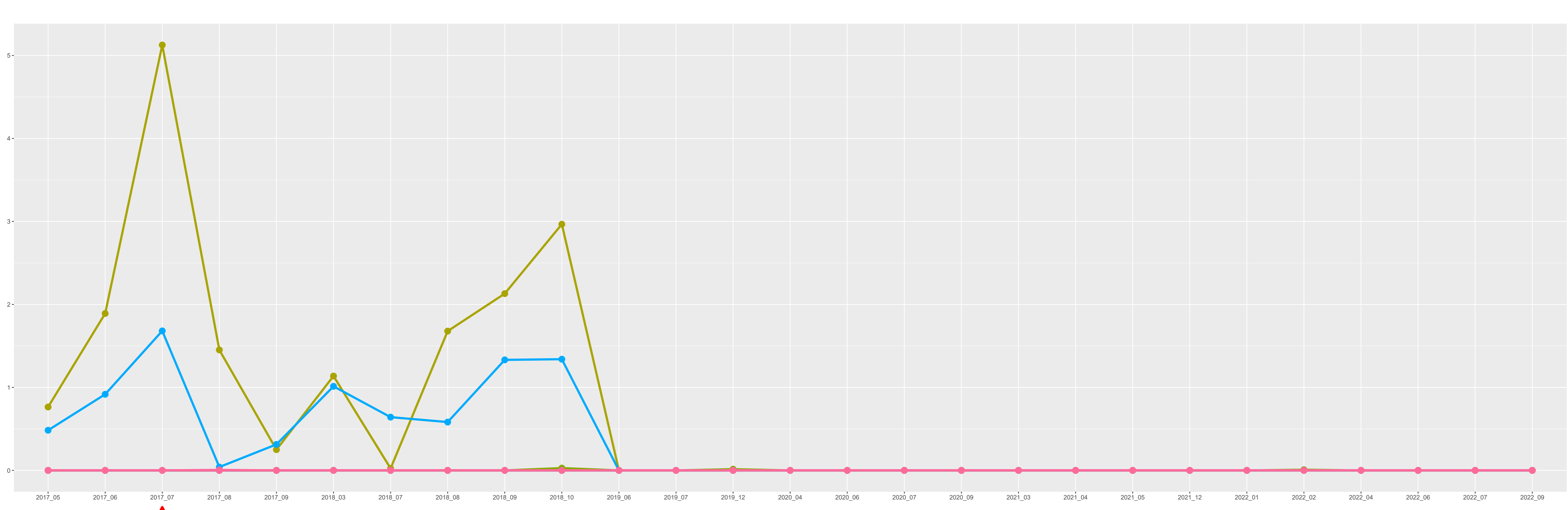

P7

Supplement: Supplementary file 1 [file Data_Sheet_1.pdf]

C

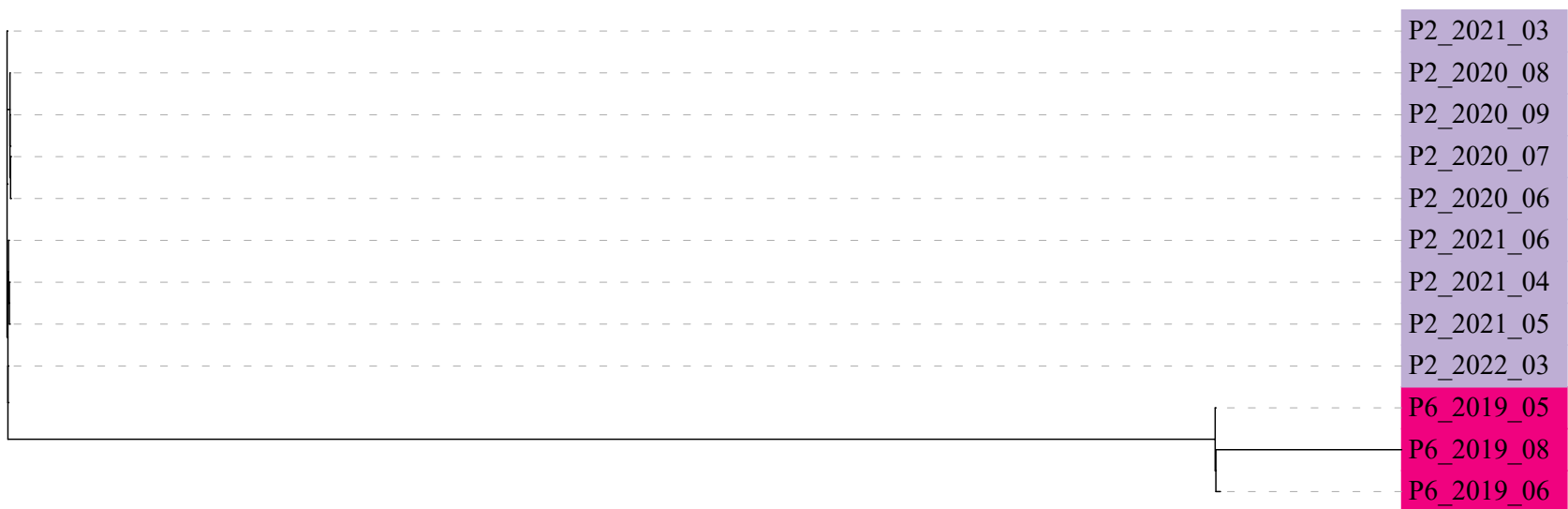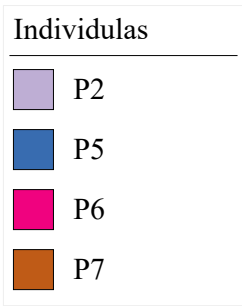

Supplement: Supplementary file 2 [file Data_Sheet_2.PDF]

# SNPdensity(SNP/kb)

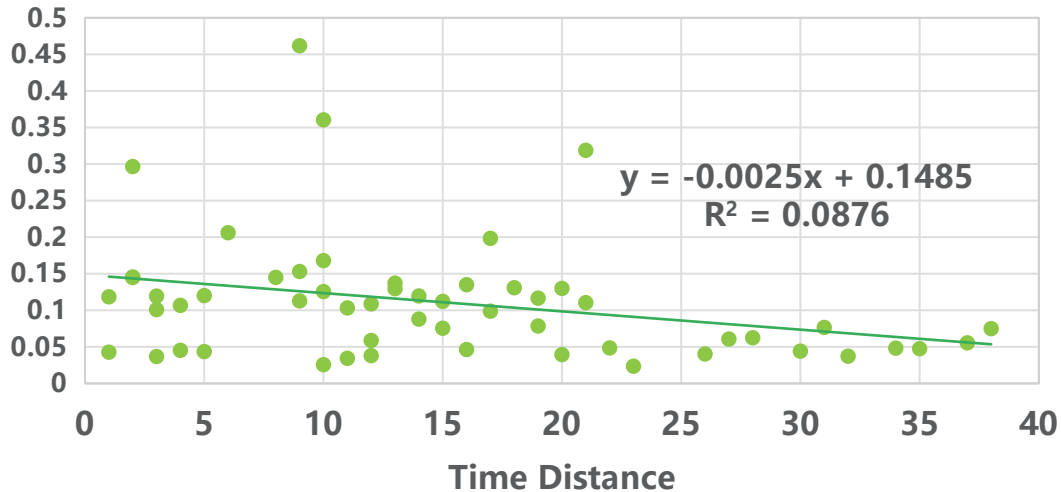

Supplement: Supplementary file 3 [file Data_Sheet_3.PDF]
